# Fellis Bovini, as a Medicine

**Published:** 1852-10

**Authors:** A. Cummings


From the Boston Med. and Surg. Journal.

*Fellis Bovini, as a Medicine.* By A. Cummings, M. D.

The bile from the gall of the ox has long been known to possess medicinal properties, and to some extent it has been used by the profession as a remedial agent, but I believe it has never gained that confidence among practitioners of which its real value renders it worthy. I have used it somewhat extensively in my practice for a few years past, and in this article I design only to give the result of my own experience with it, and the conclusions to which I have arrived in relation to its real and comparative value. Before proceeding, however, I would remark that the *form* in which I have almost immediately exhibited it, is that of pills, made of the inspissated gall, rolled in flour, magnesia, pulv. glychyrriza, or some other fine powder, to render them of a suitable consistency. The gall may be evaporated in shallow basins, in an oven, or in the sun, until it becomes sufficiently firm to form into pills as above. This, in my opinion, is far the best method. It may be given in its liquid form, but it is less agreeable to the patient, and if not mixed with proof spirits will soon become unfit for use. The medical properties of this agent, so far as my observation goes, are *laxative*, *alterative*, and *slightly tonic*. Its most valuable agency is exerted upon the stomach and liver. It seems to combine, in a remarkable degree, the three properties named above, and for many diseases to which the chylopoietic viscera are liable, I have found it a most excellent and valuable remedy. I proceed now to notice its application as a remedial agent in disease.

1st. *Obstinate Constipation.*—I am well aware that this is much oftener a symptom of disease, than disease *per se;* but I notice it in this place in order to speak of it in that form so common to those engaged in sedentary and confined occupations, and especially of females in large cities, amongst whom, for want of proper exercise and care, constipation is so prevalent and detrimental. In cases of this description, I have seldom, if ever, been disappointed of obtaining relief by the use of the agent under consideration. The hardened, compact, clay-colored fæces so common in cases where there is more or less obstruction of the liver, are, by the use of the gall, broken down in the intestines, and rendered so friable as to be easily discharged. This result is procured, not only by the combination of the gall with the hardened concretions, rendering them soft and unirritating, but also by removing the obstruction, and permitting the natural flow of bile from the gall-bladder into the intestines. Thus it answers, in this respect, a two-fold purpose. Any one who doubts the efficacy of this remedy, by pouring a few drops of fresh gall upon hard clay-colored fæces, and observing how soon the mass becomes liquid, cannot fail to be convinced. It imparts a healthy tone to the bowels, and promotes the natural secretions which may become impaired.

2d. In *bilious diseases*, arising from a torpid action of the hepatic function, the gall is an excellent remedy. It seems to act as a stimulant to the liver, and promotes the secretion of the bile, and also to cause it to flow freely into the bowels, and thus accomplish its normal functions in the animal economy.

3d. In *jaundice*, you will find that the exhibition of gall, if continued sufficiently long, even in small doses, will not fail to accomplish a desirable and satisfactory purpose. I could relate the history of many cases in my own practice, in which there was every symptom of this disease, where the gall has acted in the most satisfactory manner. As a stimulant to the liver, I generally prefer it to blue pill or mercury in any form, though there may be chronic diseases in which the mercury or some other alterative would be preferable. Whoever, at least, will thoroughly test the powers of this agent, I am confident, will find that I have not exaggerated its value. At least I am willing to abide by the judgment of others who may test it, as to the truth of my assertions. It may be necessary to continue the exhibition of this remedy for some length of time in severe cases of jaundice, but it is perfectly harmless, and moreover it may be used in those cases in which mercury cannot be administered, on account of the prejudice of patients or their friends against it, or of any idiosyncracy of constitution where its use may be interdicted. At least it is a most valuable auxiliary.

4th. In *dyspepsia*, also, I have in many cases seen the most gratifying results from the use of this remedy. It seems to impart a good tone to the stomach, and by its laxative effects upon the bowels, as well as by its soothing the irritated mucous surfaces of the stomach, proves, in my hands, at least, an excellent remedy. It leaves the influence of a mild tonic bitter on the stomach, not sufficient, however, to produce pain; and its laxative effects in dyspepsia cannot be but beneficial, for, in most cases in this disease, the bowels are torpid, and not unfrequently obstinately constipated. As a remedy also collaterally.

5th. In *hemorrhoids* and *prolapsus ani*, the gall is justly entitled to our consideration and confidence. If it has no direct or specific influence in removing these forms of disease, it is at least one of the best laxatives in the general torpor of the bowels which accompanies them, since it not only evacuates, but soothes the bowels, and does not produce the irritation in piles and prolapsus ani that most other articles of the class do. But I am also inclined, from my experience with the article, to believe that it exerts a very favorable influence, at least, in the *cure* of these troublesome and painful affections. At least it justly merits a fair trial.

6th. In *bilious and intermittent fevers*, the gall cannot but exert a favorable influence, since its office is not only to act as an alterative, and rouse the liver to its wonted action, but also to carry off from the bowels the superabundant bile, and give tone to the chylopoietic system. In a word, in all those forms of disease, (and they are many,) arising from torpor of the hepatic system, I believe that there are few medicines that will give equal satisfaction with the one under consideration, and it is certain that no remedy is more safe in its administration and effects.

One more tormenting and dreaded effect arising from bilious derangement, in which the gall acts in a very favorable manner, I had almost forgotten to mention.

7th. *Sick headache*, so called, though not strictly speaking, a disease, is a sympathetic, symptomatic affection, of very frequent occurrence, and always excruciating, and dreaded by those who are subject to it periodically, or occasionally. As it arises from a bilious state of the stomach, the gall, given in small doses, and as frequently as is necessary, seldom fails to mitigate the symptoms, or entirely to relieve, or prevent its accession. It should be given in periodic cases for a season of at least a day or two before the anticipated attack.

8th. In *typhus and typhoid fevers*, it is an excellent laxative, where strong cathartics are not required, and will be found worthy of confidence, whenever a remedy of the class seems to be indicated. Also in the low forms of

9th. *Nervous and continued fevers*, no better laxative, in my judgment, can be found, since in those cases strong cathartics are almost invariably contra-indicated. But I need not particularize further, since I believe enough has been said to give my ideas in relation to the class of cases in which this remedy is indicated; and as I cannot expect to gain the confidence of practitioners without their first giving the article in question a fair and impartial trial, I have perhaps already written too much. I am confident, however, that those who may make a fair trial of it will not accuse me of exaggeration, for I have endeavored candidly to give the value of the article as it has proved itself in my own practice, and not from theory deduced from the natural properties of the medicine. I have said it is necessary, not unfrequently, to continue the medicine for some length of time, in obstinate cases especially. But it is harmless and safe, and will act well in any constitution, and is contra-indicated by no form of idiosyncracy. The dose of the inspissated gall in the form of pills, or otherwise, is from five to ten grains or more, repeated every two or three hours, for a cathartic, and less for a mere laxative effect. It may be given in sufficient doses, at any time, with perfect safety to adults or children.

